# Hyaluronic Acid Is an Effective Dermal Filler for Lip Augmentation: A Meta-Analysis

**DOI:** 10.3389/fsurg.2021.681028

**Published:** 2021-08-06

**Authors:** László Márk Czumbel, Sándor Farkasdi, Noémi Gede, Alexandra Mikó, Dezső Csupor, Anita Lukács, Valéria Gaál, Szabolcs Kiss, Péter Hegyi, Gábor Varga

**Affiliations:** ^1^Department of Oral Biology, Faculty of Dentistry, Semmelweis University, Budapest, Hungary; ^2^Institute for Translational Medicine, Medical School, University of Pécs, Pécs, Hungary; ^3^Department of Pharmacognosy, Faculty of Pharmacy, University of Szeged, Szeged, Hungary; ^4^Department of Public Health, Faculty of Medicine, University of Szeged, Szeged, Hungary; ^5^Department of Physiology, Anatomy and Neuroscience, Faculty of Science and Informatics, University of Szeged, Szeged, Hungary; ^6^Department of Ophthalmology, Medical School, University of Pécs, Pécs, Hungary; ^7^Doctoral School of Clinical Medicine, University of Szeged, Szeged, Hungary

**Keywords:** hyaluronic acid, dermal filler, lip augmentation, effectiveness, adverse effects

## Abstract

**Introduction:** The lips and the mouth play an indispensable role in vocalization, mastication and face aesthetics. Various noxious factors may alter and destruct the original structure, and appearance of the lips and the anatomical area surrounding the mouth. The application of hyaluronic acid (HA) may serve as a safe method for lip regeneration. Although a number of studies exist for HA effectiveness and safety, its beneficial effect is not well-established.

**Aim:** The present meta-analysis and systematic review was performed to investigate the effectiveness of HA on lip augmentation. We also investigated the types and nature of adverse effects (AEs) of HA application.

**Methods:** We reported our meta-analysis in accordance with the PRISMA Statement. PROSPERO protocol registration: CRD42018102899. We performed the systematic literature search in CENTRAL, Embase, and MEDLINE. Randomized controlled trials, cohort studies, case series and case reports were included. The untransformed proportion (random-effects, DerSimonian-Laird method) of responder rate to HA injection was calculated. For treatment related AEs descriptive statistics were used.

**Results:** The systematic literature search yielded 32 eligible records for descriptive statistics and 10 records for quantitative synthesis. The results indicated that the overall estimate of responders (percentage of subjects with increased lip fullness by one point or higher) was 91% (ES = 0.91, 95% CI:0.85−0.96) 2 months after injection. The rate of responders was 74% (ES = 0.74, 95% CI:0.66−0.82) and 46% (ES = 0.46, 95% CI:0.28−0.65) after 6 and 12 months, respectively. We included 1,496 participants for estimating the event rates of AEs. The most frequent treatment-related AEs were tenderness (88.8%), injection site swelling (74.3%) and bruising (39.5%). Rare AEs included foreign body granulomas (0.6%), herpes labialis (0.6%) and angioedema (0.3%).

**Conclusion:** Our meta-analysis revealed that lip augmentation with injectable HA is an efficient method for increasing lip fullness for at least up to 6 months after augmentation. Moreover, we found that most AEs of HA treatment were mild or moderate, but a small number of serious adverse effects were also found. In conclusion, further well-designed RCTs are still needed to make the presently available evidence stronger.

## Introduction

The lips and the mouth have a crucial functional importance in vocalization and mastication. Additionally, they also play an important role in the aesthetics of the face (1, 2). Particularly, lip fullness is a key factor associated with attractiveness, beauty and youth (2, 3). A number of noxious and hereditary factors contribute to the deterioration of the perioral tissues with age (2, 4–7). Consequently, volume loss of the lips may occur with other signs of aging, such as the appearance of perioral lines, marionette lines and flattening of the cupid bow (2). There are several surgical and non-surgical reconstructive procedures aiming to restore oral competence, anatomical structures and to provide appealing aesthetic outcomes and in order to be more attractive (6, 8).

Theoretically, there is a wide range of possible reconstructive methods that can be applied to rebuild damaged tissues such as tissue engineering using stem cells (9–11), gene therapy (12, 13) and artificial biocompatible scaffolds (14, 15), but their use has been not well-established in routine clinical settings. Among the non-surgical regenerative and reconstructive procedures, hyaluronic acid (HA)-based dermal filling is one of the most frequently used treatments (16, 17). Its advantages over other filling materials include its natural occurrence, which provides non-immunogenic properties (18). It also exerts an antioxidant effect (19, 20), and anti-inflammatory activity (18, 21). Additionally, HA highly supports tissue regeneration and wound-healing by providing a suitable structure for cell ingrowth (22, 23). Due to its multiple advantageous properties, HA is also broadly used in other areas of tissue regeneration, such as orthopedics to treat osteoarthritis and rheumatoid arthritis (24, 25). Moreover, it is utilized in ophthalmology, dermatology (26), as well as in certain dental procedures (27–29).

The initial production of HA from animal sources was shifted to bacterial production. In this process, various genetically modified bacteria such as *B. subtilis* and Group A and C *Streptococci* are used to produce HA, which is then extracted and chemically further modified to create cross-links between HA polymers (30, 31). This advancement in production greatly contributed to its recent success with decreased manufacturing costs, increased purity of the products, and decreased immune reactions (32).

Since the approval of the first non-animal based HA in 2004 (8) several clinical trials aimed to reveal its true potentials. HA is believed to be an excellent candidate for soft tissue augmentation to restore lip fullness, cosmetic asymmetries and to deal with rhytids due to the loss of elasticity of connective tissue (7). However, clinical studies investigating effectiveness were conducted with small sample sizes and with short follow-up periods. Therefore, conclusions rely on weak evidence, including high levels of uncertainty.

No meta-analysis has been conducted to determine the effectiveness of HA for lip augmentation and to confirm its long-term aesthetic results. Thus, the main objective of the present meta-analysis and systematic review was to increase the power and precision of the estimated HA effect on lip augmentation. Secondarily, we investigated the number and nature of adverse effects (AEs) of HA published in the literature.

## Materials and Methods

### Protocol and Registration

This meta-analysis was reported in accordance with the Preferred Reporting Items for Systematic Reviews and Meta-Analyses (PRISMA) Statement (33) using similar approaches that we have recently reported (34–36). The PRISMA checklist summarizing the content of this review is enclosed in the supporting information (Supplementary Table 1). The meta-analysis was registered in PROSPERO (International Prospective Register of Systematic Reviews), 10/12/2018, Registration Number: CRD42018102899. There were no deviations from the study protocol.

### Eligibility Criteria

The PICO (patient characteristics, type of intervention, control, and outcome) format was applied to investigate the following clinical questions: (1) To what extent are hyaluronic acid dermal fillers effective for lip augmentation? (2) What are the common and also the rare treatment-related adverse effects of HA application?

For analysis, we considered records published in scientific journals meeting the requirements of our selected PICO. Patient characteristics: subjects above 18 years having a minimal, mild or moderate score on a validated lip fullness scale. Type of intervention: injecting hyaluronic acid dermal filler into the lips and perioral area to increase lip fullness and enhance aesthetic appearance. Control: base-line control—baseline values of lip fullness recorded before treatment. Lip fullness values recorded after treatment were compared to baseline values. The effectiveness was evaluated as the rate of responders. A responder was defined as a participant with at least one grade improvement on a validated lip fullness scale. Outcome, primary: effectiveness measurement, i.e., the number of responders at each check-up; secondary: number and type of AEs related to treatment.

### Inclusion and Exclusion Criteria

Publications which met the following eligibility criteria were included: (1) randomized controlled trials, cohort studies or case series and case reports; (2) intervention: hyaluronic acid used for lip augmentation; (3) healthy adult participants; (4) records written in English or available in English translation; (5) site of injection: lips; (6) use of validated scale to measure outcome. Exclusion criteria were: (1) filling material other than hyaluronic acid; (2) site of injection other than lips and perioral area; (3) Previous facial surgery, permanent facial implants or any facial cosmetic procedure in the last 24 months.

### Information Sources and Search

A systematic search limited to English language records was performed in three different major electronic databases [Cochrane Central Register of Controlled Trials (CENTRAL), Embase and MEDLINE (*via* PubMed)] on 31 December, 2018. Besides electronic databases, an extensive hand search in the reference list of relevant articles and included records were also performed to find eligible records. Gray and black literature was not considered for this meta-analysis. “Hyaluronic acid” and “lip” search terms and their synonyms were used in each database adapted to their specific search engines. Supplementary Table 2 contains the detailed search quey.

### Study Selection

The EndNote (Clarivate Analytics, Philadelphia, US, version: X9.3.3) reference manager was used to organize and manage records. After removing duplicates, the remaining records were screened for suitability by two authors (L.M.C. and S.F.), in duplicate, based on the titles and abstracts of the published original papers. The eligibility of full texts of the remaining records was assessed by the same two review authors independently. Disagreements between reviewers were resolved by discussion or, if it was necessary, by consulting a third review author (G.V.).

### Data Collection Process and Data Items

Data extraction was performed by two authors independently (L.M.C. and S.F.) using a preconstructed standardized data extraction form. The following information was extracted: first author's name, year of publication, sample size, age and gender distribution, study design, type of HA used, site of injection, follow-up period, type of validated scales used for evaluation, outcome (rate of responders, number and type of AEs). In case of disagreement, a third author (G.V.) was also involved.

### Risk of Bias Assessment

Quality and risk of bias of the RCTs were evaluated by two authors (L.M.C. and S.F.) independently. Assessment was based on the recommendation of the Cochrane Collaboration, the Cochrane Risk of Bias assessment tool (37, 38). In case of disagreement a third author was involved (G.V.). Studies were evaluated according the domains specified in the Cochrane Handbook for Systematic Reviews of Interventions (38).

Cohort studies were evaluated based on the Newcastle Ottawa Scale (NOS) for Cohort Studies (39). We slightly modified the original NOS scale. We removed “Ascertainment of exposure” subdomain from Selection domain. Thus, in the Selection domain three sub domains remained: “Representativeness of the exposed cohort,” “Selection of the non-exposed cohort,” and “Demonstration that outcome of interest was not present at start of study.” Scores for these subdomains were given according to the original NOS scale (39). Hence the maximum score was three, one and three stars for Selection, Comparability and Outcome domains, respectively. In the outcome 6 months or more of follow-up was considered acceptable. Drop-out below 10% was considered adequate. Supplementary Table 3 summarizes the modified NOS.

### Summary Measures and Synthesis of Results

Untransformed proportions with 95% confidence intervals (CIs) were calculated for the rate of responders. A responder is defined as a participant with at least one grade improvement on a validated lip fullness scale compared to its baseline value. For analyzing AEs we used descriptive statistics, summing the sample sizes of included studies and the incidence of each AEs described in any of the included publications. The number of participants was chosen as statistical unit.

We only considered results credible if raw data for meta-analysis could be drawn from at least three records. We applied the random effect model with DerSimonian-Laird method. *I*^2^ and chi-square tests were used to quantify statistical heterogeneity and gain probability-values, respectively; *p* < 0.1 indicated significant heterogeneity (38). All statistical analyses were performed using STATA 15.0.

### Publication Bias

We constructed funnel plots and performed visual inspection of their results to check for publication bias.

### Certainty of Evidence Pont

The GRADE approach was followed to evaluate the quality and certainty of evidence (37, 40). Assessment was performed independently by two review authors (L.M.C and S.F.).

## Results

### Study Selection

**Figure 1:**
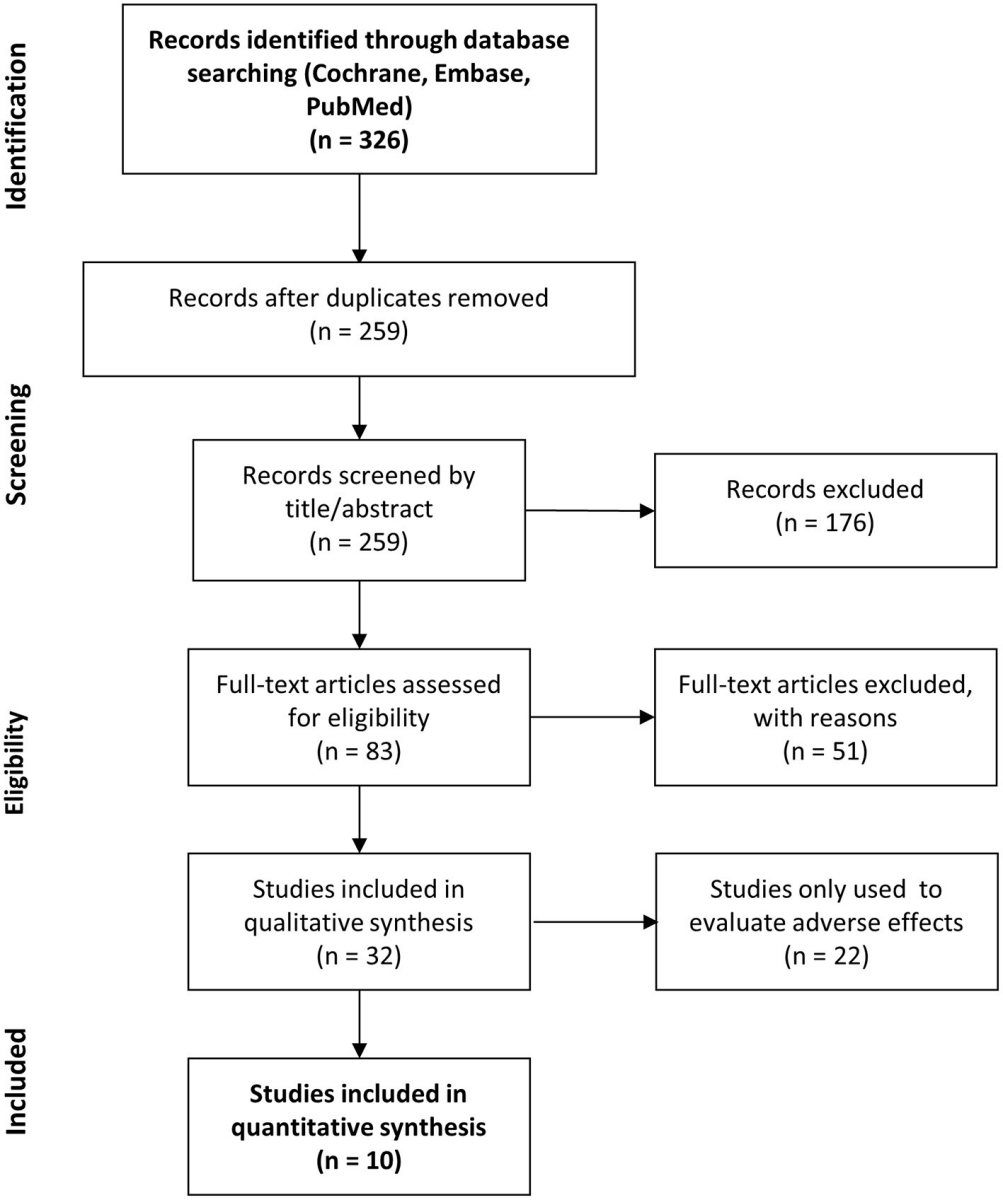
PRISMA flow chart. Summarizing the study selection process.

During the study selection process, we identified a total of 326 records. After removing duplicates, 259 items remained. During the screening process, 176 records were excluded due to various reasons such as filler material other than HA (*n* = 30) or different injection sites (*n* = 7), focusing on novel methods of injection (*n* = 24), investigating the effect of hyaluronic acid in special implications outside the scope of this meta-analysis (*n* = 64), review articles (*n* = 17) or miscellaneous (*n* = 34). Afterwards, 83 full text records were searched. Out of these publications, 32 were included in the qualitative synthesis and 10 in the quantitative synthesis assessing the effectiveness of lip augmentation (Figure 1).

### Study Characteristics

#### Description of the Included Studies

We included 5 RCTs (41–45) and 5 cohort studies (46–50) to analyze the effects of HA on lip augmentation. Two additional RCTs (51, 52), six additional cohort studies (53–58) and 14 case reports (59–72) were included for assessing AEs.

In the effectiveness analysis, a total of 1,228 participants were included. Subjects aged 18 or older desiring lip augmentation, had lip fullness of minimal, mild or moderate on a validated lip fullness scale were included. In the study population all Fitzpatrick skin types have been represented. Exclusion criteria included a history of allergy to injectable HA, history to any semi-permanent or permanent tissue augmentation or aesthetic surgery or any temporary dermal filler treatments in the last 24 months in the facial region. Subjects with scarce or significant abnormalities of the lips were also excluded. The mean age of subjects in the studies varied between 41 and 54 years. Altogether 4 different injectable HA products were used, Juvéderm and Restylane were the two most commonly applied ones. Follow-up periods varied between 12 and 48 weeks. All included records utilized a validated lip fullness scale such as the Medicis Lip Fullness Scales (73) or the Allergan Lip Fullness Scale (74).

AEs were collected and assessed from a total of 32 records including 1,488 participants and more than 12 different HA products. In all includes studies the lips and perioral lines were the site of injection. A tabulated summary of the characteristics of the included studies and HA products is provided in Tables 1–3 and Supplementary Table 4.

**Table 1:** Study characteristics of records included in effectiveness analysis.

**References**	**Study design**	***n*** ^*^	**Female** **ratio**	**Age: mean ± SD (median) (range)**	**Intervention**	**Control**	**Maximum injected volume; injection technique**	**Follow-up (months)**	**Outcome measure**
Beer et al. (41)	RCT, multicentre, evaluator blinded	199	97%	45.5	Restylane-L	No-treatment group	2.17 ml (mean); anterograde, retrograde linear threading, serial puncture	6	MLFS and WASULL, GAIS, TEAEs
Chopra et al. (46)	Cohort, multicentre, open label, prospective	57	93%	46.5, (23–72)	Restylane-L	Baseline-controlled	1–3 ml (range); submucosa, retrograde, anterograde linear, fanning	3	GAIS, MLFS, TEAEs
Dayan et al. (42)	RCT, multicentre, evaluator blinded	208	95.8%	(49), (20–79)	Juvéderm Ultra XC (HYC-24L)	No-treatment group	4.8 ml (max); linear threading, serial puncture, fanning, crosshatching	12	ALFS, POL, OCS, ISRs, AEs
Eccleston et al. (47)	Cohort, multicentre, open label, prospective	59	100%	50, (21–74)	Juvéderm Volbella	Baseline-controlled	1.3 ml (median); retrograde, tunneling, crosshatching	12	ALFS, AEs
Fagien et al. (48)	Cohort, multicentre, evaluator blinded, prospective	50	96%	(47), (24–68)	Juvéderm Ultra	Baseline-controlled	2.2 ml (median), 2.3 ml (max); retrograde, anterograde, tunneling, serial puncture	12	ALFS, OCS, POL, CTR, AEs
Geronemus, et al. (43)	RCT, multicentre, evaluator blinded	224	96.9%	(54), (22–78)	Juvéderm Volbella XC (VYC-15L)	Restylane-L	2.5 ml (median); subdermal, intradermal, tunneling, puncture	12	ALFS, POLSS, POLM, OCS, GAIS, AEs
Glogau et al. (44)	RCT multicentre, evaluator blinded	135	99%	47.6 ± 10.6, (50.0), (18.0–65.0)	Restylane	No-treatment group	1.5 ml (max), 0.3–2.5 ml (range); linear injection technique, serial puncture	6	MLFS, GAIS, AEs
Raspaldo et al. (45)	RCT multicentre, evaluator blinded	268	97.1%	(48), (18–76)	Juvéderm Volbella (with Lidocaine)	Restylane-L	1.97–1.86 ml (mean); intradermal, subdermal, tunneling	12	ALFS, POL, OCS, AEs, ISRs,
Solish and Swift (49)	Cohort, multicentre, evaluator blinded, prospective	18	86%	41.1 ± 11.4, (40), (26–65)	Restylane	Baseline-controlled	1.5 ml (max); anterograde, vertical, deposition formation	3	MLFS, GAIS, AEs
Yazdanparast et al. (50)	Cohort, single center, open label, prospective	10	100%	(28–45)	Hyamax Kiss	Baseline-controlled	1 ml (max); retrograde	6	MLFS, IGA, VAS, AEs

* *Number of participants included in the MA analysis (Exclusion due to study groups using different filling material or other anatomical sites.)*.

*AEs, Adverse events; ALFS, Allergan Lip Fullness Scale; CTR, common treatment-site responses; GAIS, Global Aesthetic Improvement Scale; IGA, Investigator's Global Assessment; ISRs, Injection site responses; MLFS, Medicis Lip Fullness Scale; OCS, Oral Commissure Severity Scale; POL/POLSS, Allegran Perioral Severity Scale; POLM, Allergan Perioral Lines at Maximal Contraction scale; SP, Standardized photography; TEAE, treatment-emergent adverse events; VAS, Visual Analog Scale; WASULL, Wrinkle Assessment Scale of Upper Lip Lines*.

**Table 2:** Study characteristics of RCTs and cohort studies only included in adverse effect analysis.

**Study**	**Study design**	***n*** ^*^	**Female ratio**	**Age: mean ± SD (median) (range)**	**Intervention**	**Control**	**Statistics**	**Follow-up (weeks)**	**Outcome**
Artzi et al. (53)	Cohort, multicenter, retrospective	3^†^	90%	49.6, (28–70)	Juvéderm Volbella (Allergan)	No control group	Spearman correlation	96	Immediate and delayed AEs
Carruthers et al. (54)	Cohort, single center, open label	15	100%	(40.50), (33–60)	Restylane	No control group	Descriptive statistics	24	SP, AEs
Carruthers et al. (52)	Randomized, parallel-group, multicentre, clinical trial	23	100%	48.4 ± 5.5	Juvéderm Ultra, Juvéderm Ultra Plus	OnabotulinumtoxinA, OnabotulinumtoxinA plus hyaluronic acid	Kruskal-Wallis test, Wilcoxon rank sum test	24	GAIS, CIS, AEs
Downie et al. (51)	Randomized, parallel-group, double blinded, single-center, clinical trial	23	100%	(25–55)	Perlane	Various collagen fillers	Kruskal Wallis Rank Sum test	48	2D and 3D facial image analysis, AEs
Fischer et al. (55)	Cohort, multicenter, retrospective	146	98.6%	44.7 ± 14.6	CPM-HAL1 and CPM-HAL2 (Belotero Balance Lidocaine)	No control group	Descriptive statistics	16	Merz scale, GAIS, VAS, AEs
Philipp-Dormston et al. (56)	Cohort, multicenter, open label, prospective	60	88.7%	39.7 (21–75)	Juvéderm Volbella	No control group	Descriptive statistics	4	4-grade scale for subject and injector satisfaction, AEs
Rzany et al. (57)	Cohort, multicenter, open label, prospective	76	94.8%	54.5 ± 8.2	Emervel	No control group	Descriptive statistics	24	GAIS: LRS, LFGS, satisfaction questionnaires, AEs
Samuelson et al. (58)	Cohort, multicenter, evaluator blinded, prospective	29	100%	36, (19–59)	Restylane Lip Volume	Baseline-controlled	Proportion with 95% CI	36	GAIS, MLFS, AEs

* *Number of participants included in the MA analysis (Exclusion due to study groups using different filling material or other anatomical sites*.

† *Study population number is 400 (mean age: 49.6, range: 28–70), however only 3 patients received lip augmentation with HA filler*.

*AEs, Adverse events; CIS, Cosmetic Improvement Scale; GAIS, Global Aesthetic Improvement Scale; ISRs, Injection site responses; LFGS, Lip Fullness Grading Scale; LRS, Lemperle Rating Scale; MLFS, Medicis Lip Fullnes Scale; RCT, Randomized controlled trial; SP, Standardized photography; VAS, Visual Analog Scale*.

**Table 3:** Characteristics of hyaluronic acid dermal fillers assessed in the analysis.

**Product name**	**Concentration**	**Composition**	**References**	**Source of information**
Belotero intense lidocaine	25 mg/ml	Cross-linked	Fischer et al. (55)	(75)
Emervel (range of products)	20 mg/ml	Cross-linked to various degree	Rzany et al. (57)	(57)
Hyamax Kiss	22 mg/ml	500 μm particle size, cross-linked	Yazdanparast et al. (50)	(50)
Juvéderm Ultra	24 mg/mil (0.3% Lidocaine)	Cross-linked (6%)	Fagien et al. (48); Carruthers et al. (52)	(48, 54)
Juvéderm Ultra XC (HYC-24L)	24 mg/mil (0.3% Lidocaine)	Cross-linked	Dayan et al. (42); Bulam et al. (60)	(42)
Juvéderm Volbella without Lidocaine	15 mg/ml	Not available	Eccleston et al. (47); Artzi et al. (53)	(47)
Juvéderm Volbella with Lidocaine	15 mg/ml HA (0.3% Lidocaine)	Cross-linked	Raspaldo et al. (45); Philipp-Dormston et al. (56)	(45, 76)
Juvéderm Volbella XC (VYC-15L)	15 mg/ml HA (0.3% Lidocaine)	Cross-linked, low- and high-molecular-weight HA	Geronemus et al. (43)	(77)
Perlane	20 mg/ml	Cross-linked, 1,000 μm particle size	Downie et al. (51)	(51, 78)
Restylane (without lidocaine)	Not available	SGP, 300 μm particle size, cross-linked	Glogau et al. (44); Solish and Swift (49); Carruthers et al. (54); Fernández-Aceñero Ma et al. (68); Anatelli et al. (59); Curi et al. (61); Dougherty et al. (62); Leonhardt et al. (70); Wolfram et al. (72); Farahani et al. (66); Edwards et al. (64)	(44, 49)
Restylane-L	20 mg/ml HA (0.3% Lidocaine)	SGP, cross-linked	Raspaldo et al. (45); Geronemus et al. (43); Beer et al. (41); Chopra et al. (46)	(41, 43, 45, 46, 79)
Restylane lip volume	20 mg/ml HA (0.3% Lidocaine)	Cross-linked	Samuelson et al. (58)	(58)
HA not further specified	N/A	N/A	Duhovic and Duarte-Williamson (63); Eversole et al. (65); Feio et al. (67); Grippaudo et al. (69); Martin et al. (71)	N/A

*N/A, not applicable; HA, hyaluronic acid*.

#### Description of Excluded Studies

During full-text analysis, we excluded 51 records. Seven RCTs, nine cohort studies, 11 case reports and nine review articles. Three records had non-English texts. Additionally, in 11 cases we found no full text to the records and 1 record was a non-interventional study. Out of the 32 articles included in the analysis of AEs, we excluded 22 publications from the effectiveness analysis. Several excluded articles did not report sufficient information on effectiveness, while the others used incomparable scales to measure the effectiveness of lip augmentation.

#### Risk of Bias Within Studies

**Figure 2:**
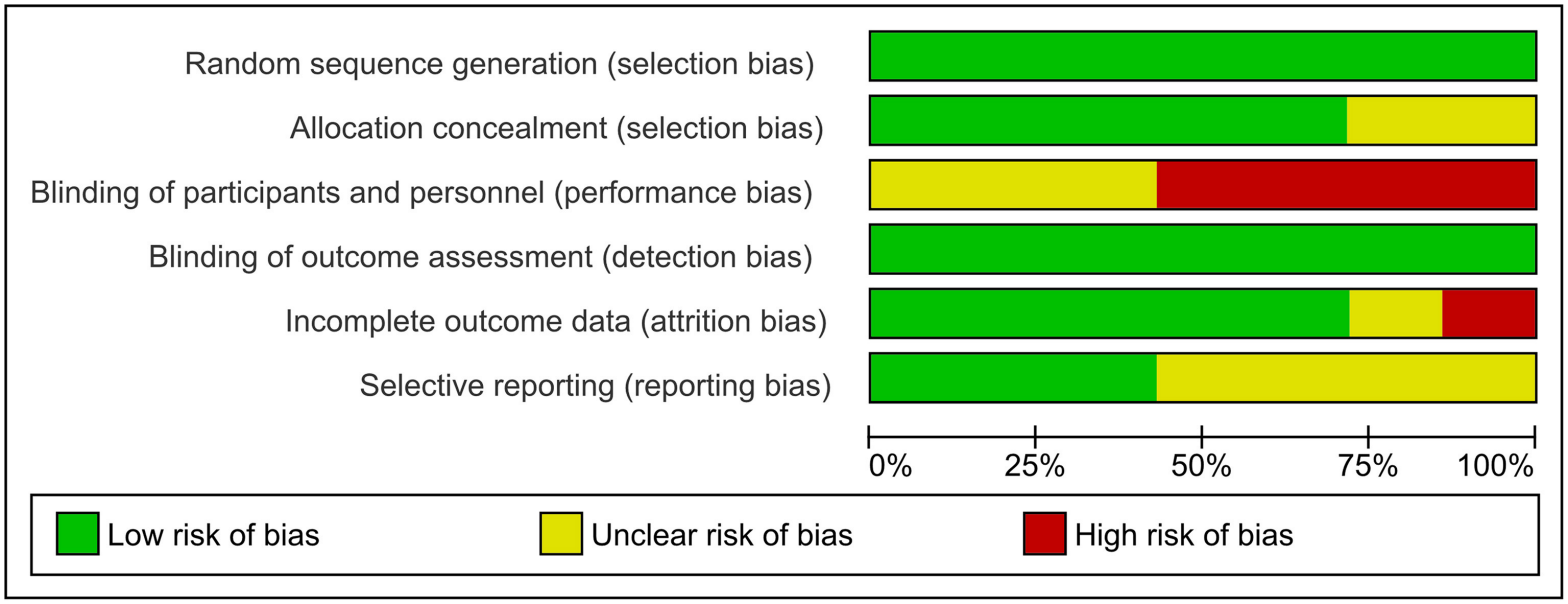
Risk of bias graph. Representing the portion of bias in each domain.

All RCTs applied means of random sequence generation. However, in the case of Carruthers et al. (52) and Dayan et al. (42) the methods used for allocation concealment were not clearly described. Due to the nature of the intervention, none of the studies applied blinding of personnel. On the other hand, the outcome assessment was performed by blinded evaluators in all studies. In the case of one study (42) attrition bias was unclear due to ambiguous reporting on lost to follow-ups. In another study (52) we found a high risk of attrition bias due to the 23% of dropouts. The level of reporting bias was low in all studies except three. Study protocols for Beer and coinvestigators (41), Carruthers et al. (52) and Glogau et al. (44) were not found. However, no intext evidence of reporting bias was found. Figure 2, Supplementary Figure 1, and Supplementary Table 5 contain the summary of the risk of bias assessment of the RCTs.

Bias in the observational studies was assessed based on the Newcastle Ottawa Scale (39). Observational studies did not have control groups. Instead, they measured the rate of responders only within the treatment group (baseline controlled). The average bias assessment score of the studies was 5.5 ± 1.3 stars on the modified seven-point scale. All 11 publications, earned three stars for selection (46–50, 53–58). Five studies received no starts for comparability (53–57). In three studies (54, 56, 58) the outcome assessments were only self-reports. One study (56) was considered to have inadequately short follow-ups for valuable results, while three studies (53, 55, 56) did not give any explanation for drop-outs. Supplementary Tables 6, 7 show the summary of the risk of bias assessment of observational studies.

### Results of Individual Studies and Their Synthesis

#### Hyaluronic Acid Treatment Effectively Increases Lip Fullness

**Figure 3:**
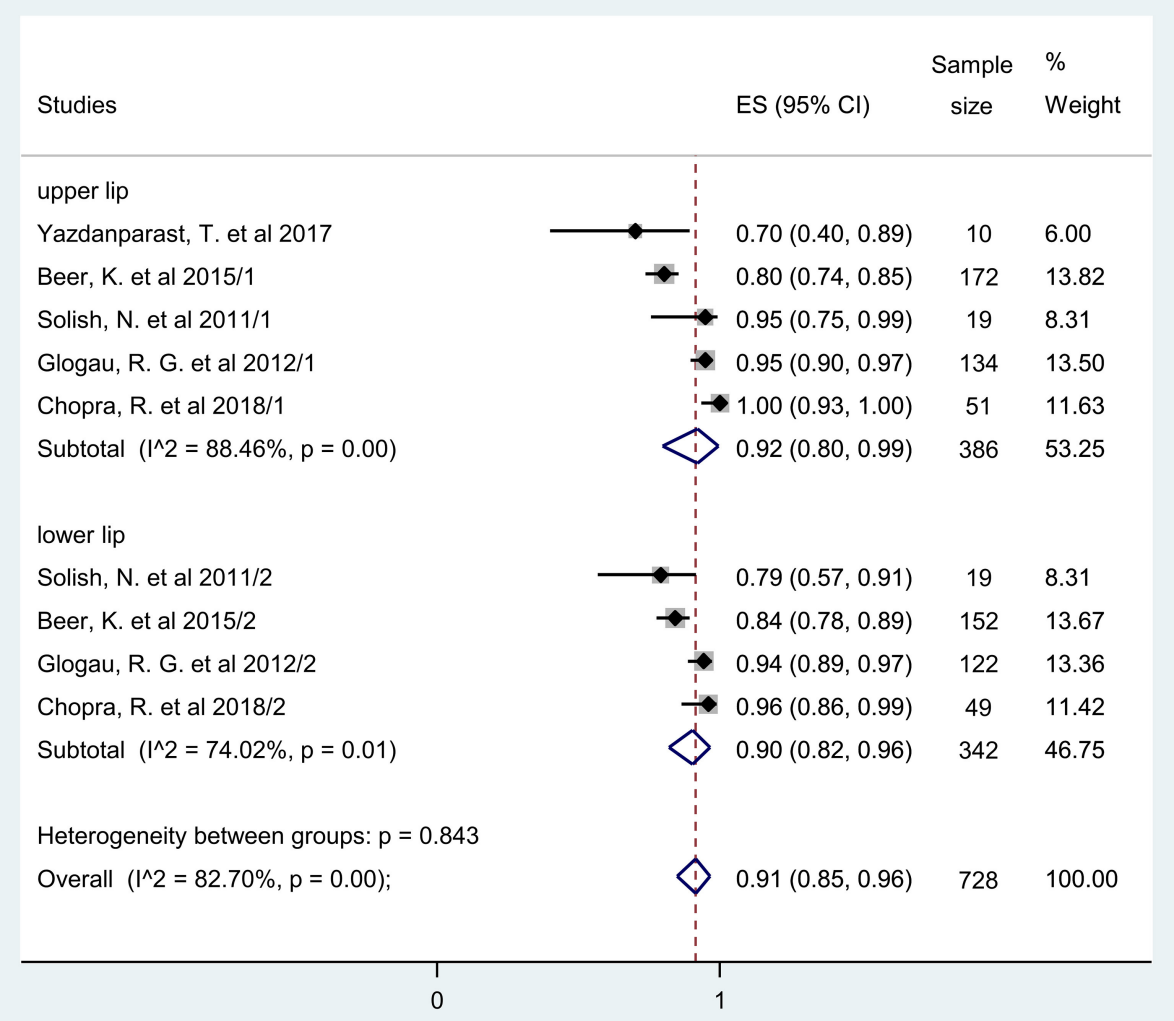
Estimate of rate of responders at 2 months after treatment for the upper and lower lips. Overall, 92% (95% CI: 80–99%) and 90% (95% CI: 0.82–96%) of included participants had at least one grade improvement on a validated lip fullness scale regarding their upper and lower lips, respectively, after 2 months of initial treatment.

Two months after HA injection the overall pooled rate of responders, i.e., the percentage of participants with at least one grade improvement on a validated lip fullness scale [Medicis Lip Fullnes Scale (MLF) or Allergan Lip Fullness Scale (ALFS)] was 91% (95% CI: 0.85−0.96) (untransformed proportion, random-effects DerSimonian-Laird method). *I*^2^-values indicating statistical heterogeneity was 82.7% (*p* = 0.0). Data were pooled from 5 studies (41, 44, 46, 49, 50) (Figure 3).

When the rate of responders for volume increase in the upper and lower lips were compared, only a very minor, 2% difference was observed between them 2 months after HA application (41, 44, 46, 49, 50). Upper lips: ES = 0.92, 95% CI: 0.88−0.99; *I*^2^ = 88.46%, *p* = 0.00 and lower lips: ES = 0.90, 95% CI: 0.82−0.96; *I*^2^ = 74.02%, *p* = 0.01 (Figure 3).

**Figure 4:**
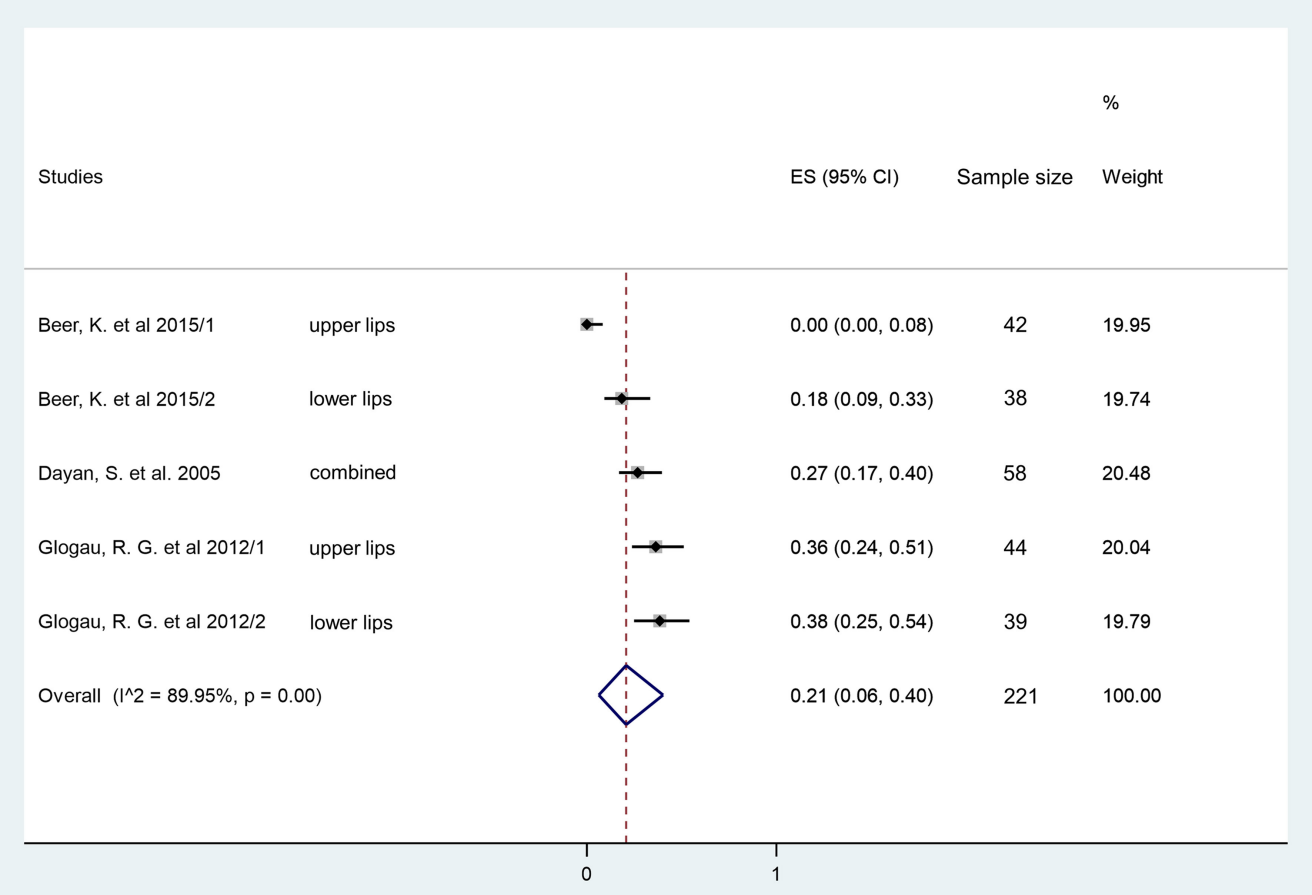
Estimate of overall rate of responders at 2 months in the no treatment group. Overall, 21% (95% CI: 6–40%) of included participants had at least one grade improvement on a validated lip fullness scale after 2 months in the no treatment group.

An additional analysis was also performed using data of the three available studies (41, 42, 44) investigating lip fullness augmentation in non-treated controls. Even among these subjects, who received no HA injection, 21% were demonstrated to be responders 2 months after baseline assessment indicating a possible placebo effect in HA injection studies (ES = 0.21, 95% CI: 0.06−0.40; *I*^2^ = 89.95%, *p* = 0.00) (Figure 4).

**Figure 5:**
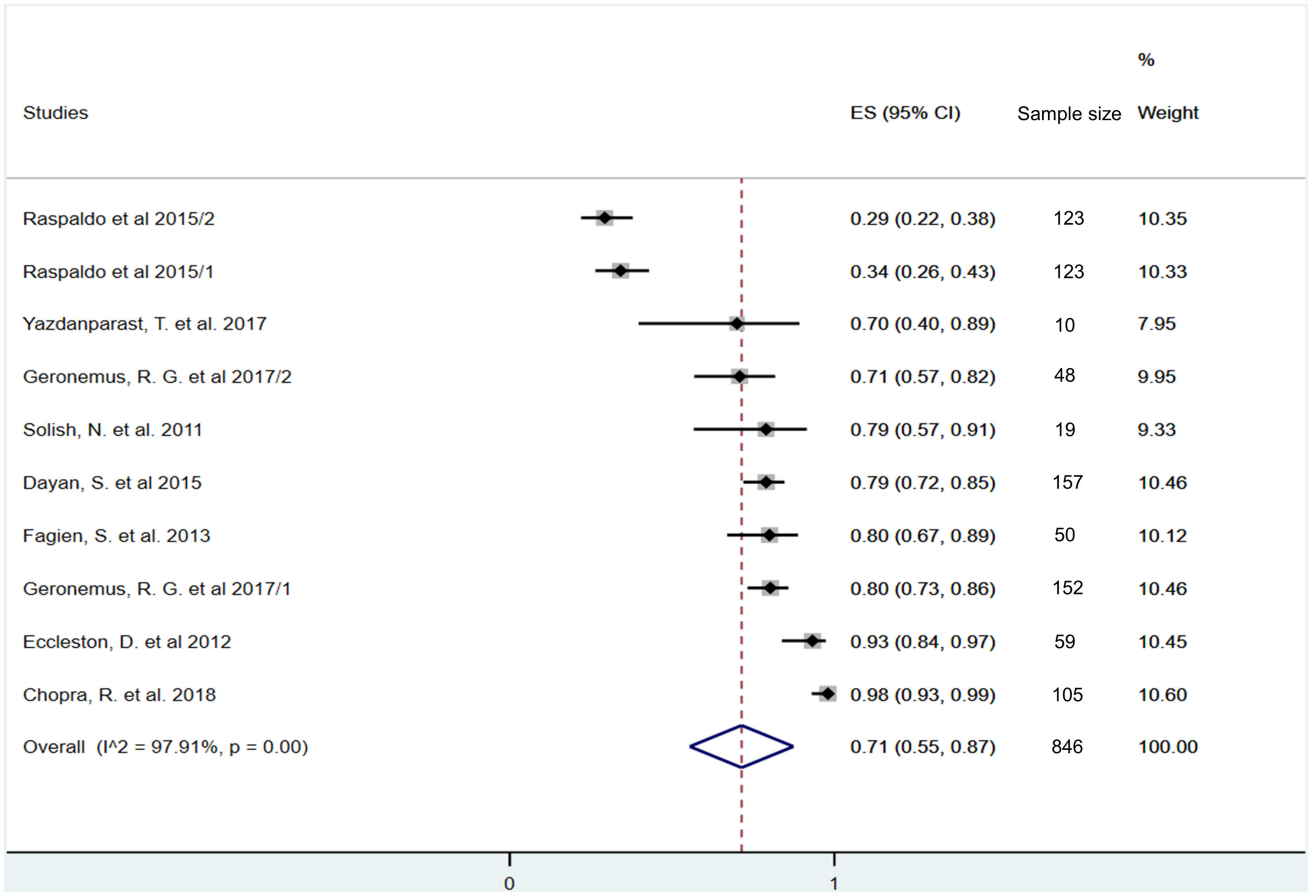
Estimate of overall rate of responders at 3 months after treatment. Overall, 71% (95% CI: 55–87%) of included participants had at least one grade improvement on a validated lip fullness scale after 3 months of initial treatment.

The rate of responders to HA treatment, i.e., the percentage of participants with at least one grade improvement on the MLF or ALF scales after 3 months, was also calculated including eight studies (42, 43, 45–50). The untransformed proportion (random-effects DerSimonian-Laird method) of the pooled data showed that 71% of the HA-treated participants were responders, meaning that 71 out of 100 experienced a substantial, at least one grade increase in lip fullness 3 months after the initial treatment (ES = 0.71, 95% CI: 0.55−0.87; *I*^2^ = 97.91%, *p* = 0.00) (Figure 5).

**Figure 6:**
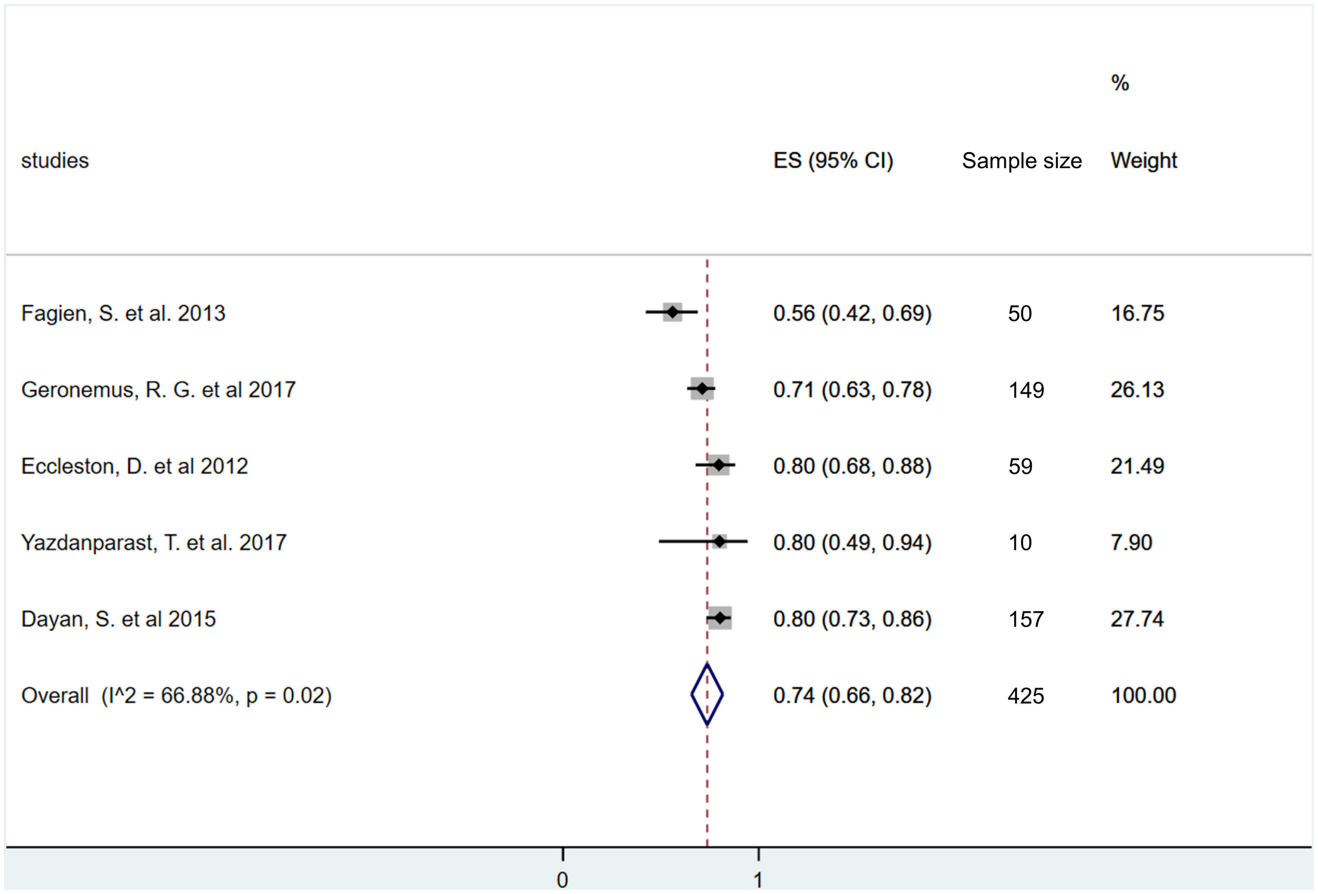
Estimate of overall rate of responders at 6 months after treatment. Overall, 74% (95% CI: 66–82%) of included participants had at least one grade improvement on a validated lip fullness scale after 6 months of initial treatment.

Six months after the HA injection, the overall rate of responders, i.e., again the percentage of those who still have an increase of lip fullness scale by one grade or higher, were synthetized from five studies (42, 43, 47, 48, 50). This analysis revealed that 74% of those who received the one dose HA treatment maintained their increase of lip volume (ES = 0.74, 95% CI: 0.66−0.82; *I*^2^ = 66.88%, *p* = 0.02) (Figure 6).

**Figure 7:**
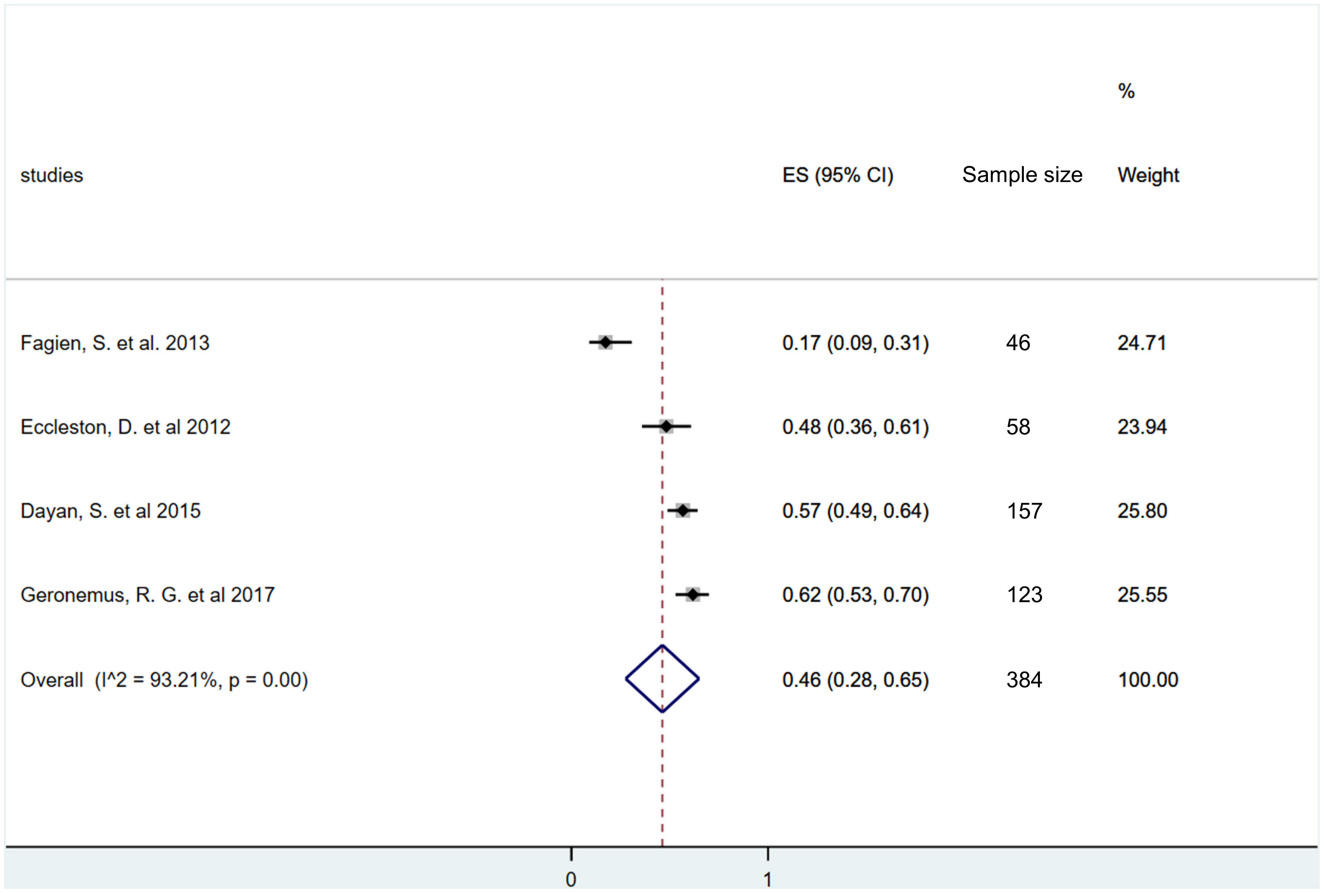
Estimate of overall rate of responders at 12 months after treatment. Overall, 46% (95% CI: 28–65%) of included participants had at least one grade improvement on a validated lip fullness scale after 12 months of initial treatment.

The lip volume data 12 months after HA application were available only in four studies (42, 43, 47, 48). Our meta-analysis revealed that rate of responders was 46% even after 1 year of a single HA injection (ES = 0.46, 95% CI: 0.28−0.65; *I*^2^ = 93.21%, *p* = 0.00) (Figure 7).

#### Adverse Effects of Hyaluronic Acid Injection

Studies reporting the AEs related to HA injections were included in this analysis. Data were pooled from six RCTs (41–43, 45, 51, 52), 11 cohort studies (46–50, 53–58) and 14 case reports (59–72) including 1,488 participants overall.

The results revealed that the five most common AEs were tenderness (*n* = 1,320, 88.7%), injection site swelling (*n* = 1,105, 74.3%), contusion (*n* = 725, 48.7%), injection site mass (*n* = 406, 27.3%), and injection site pain (*n* = 293, 19.7%). The appearance of herpes labialis (*n* = 9, 0.6%) was identified in a few cases, while filler-associated necrosis of the lips was also found very rarely in case reports. More serious AEs such asgranulomatous foreign body reaction (*n* = 9, 0.6%), were infrequent. Life-threatening angioedema was reported only in four cases out of the 1,488 patients (0.3%) included in the studies on HA injection into the lip (Supplementary Table 8).

### Publication Bias

Funnel plot constructed from studies with 3 months follow-up shows asymmetry of published records suggesting small-study effect (Supplementary Figure 2). Due to the small number of studies included, no further statistical analysis could be performed to test for small-study effect.

### Certainty of Evidence

The assessement based on the GRADE approach revealed that the final level of evidence for the effectiveness of HA treatment on lip augmentation is very low. This is explained by some low level of study designs (cohort studies), the significant inconsistency due to statistical heterogeneity indicating confounding factors, imprecision indicated by wide range of confidence intervals and suspected publication bias due to small study effect.

In the case of EAs the level of evidence is also very low due to study design, the high risk of bias and the lack of consistent reporting on EAs (Supplementary Table 9).

## Discussion

### Summary of Evidence

As it is the entrance of the gastrointestinal tract, the health and esthetics of lips are important for the well-being of the human body. Although HA is a frequently used dermal filler for non-surgical aesthetic treatment (8, 17), its benefits and possible AEs for lip augmentation have not been assessed quantitatively by meta-analysis. Although several primary studies existed on the matter, their relatively small sample size did not allow to draw strong conclusions. Our study is the first meta-analysis to integrate the available data from individual primary studies for the effectiveness of HA for lip augmentation after HA injections. In our analysis, we included studies which used validated scales to assess changes in lip fullness. We also included case reports to find-long term and rare events of treatment-related AEs. We found that HA injection effectively increases lip fullness up to 6 months among the majority of treated patients. Moreover, our analysis revealed that approximately half of the successfully treated participants still had a significantly increased lip fullness after 12 months. Most AEs related to the treatment were consistent across prospective studies. AEs were mostly mild or moderate, but rare severe AEs could also be observed in a very small number of cases.

Although our meta-analysis clearly showed the effectiveness of HA injection on the lip, the variability of the individual studies was also very obvious (41–45). This heterogeneity suggests that there were significant confounding factors that might influence the outcome of HA treatments. Several factors have been suggested to influence the outcome of lip augmentation, such as the injected volume, the number of touch-up treatments, the type of injection technique, the number of cross-links in HA product, and also the skin type of the patients, the experience of investigators, as well as the evaluation method (42, 44, 45, 80). In our analysis of effectiveness, more than 8 HA products using 5 different HA concentrations were included. The different papers reported several injection techniques and various injection volumes. Due to the high variability and the low number of studies containing identical subgroups, it was not possible to perform a comprehensive statistical analysis to investigate the effects of such confounding factors. Raspaldo and coinvestigators found that live assessment yielded more precise results compared to photo analysis based on 3D images. They argued that photographs can alter shadows and smaller rhytids, thereby altering evaluation outcomes (45). On the contrary, Moragas et al. argued that the use of a validated scale is most appropriate for evaluating lip augmentation outcomes. Yet, in their review, they suggested that anthropometric measures were far from being perfect. Therefore, they did not evaluate natural appearance or changes in the shape of the lips (80).

An important observation of our analysis is the considerable decrease in lip fullness over a 12-month period as HA treatment remained effective in only about the half of the treated patients after 1 year. Although HA is regarded to be a temporary filler, its longevity on lip volume have not been investigated with statistical methods. The decreased number of augmented lips at 12 months follow-up period could be ascribed to the natural biodegradation of HA (26). Cross-linking slows down the biodegradation of HA (81), but it is unclear to what level the concentration and degree of cross-links of HA affects its long-term effectiveness.

Unfortunately, no studies are available on the effectivness of hydratization of the lips in response to hyaluronic acid treatment. HA fillers were described to increase not only the volume, but also hydratize the treated tissue when applied (82). Namely, Seok and coinvestigators observed increased skin hydration levels after HA injections into various parts of the face (83). For AEs, we found that similar event rates were reported from the included RCTs (41–45) and other prospective studies (46–50). However, case reports revealed additional AEs (59–72), which were not reported in clinical trials. Our analysis revealed that the most frequent AEs were injection-related, such as tenderness, injection site swelling, bruising, injection site mass, injection site pain. All of these AEs resolved without the need of treatment within a few weeks. Similar AE rates were found in earlier studies for lips (3, 80, 84) and for other anatomical sites as well (8, 17).

Remarkably, in one study (51), herpes labialis was found to be the most common AE (17%). The reason for this is unclear since the prevalence of this viral infection was much lower in the other included studies (0.6%). Most probably, in the work of Downie et al. the needle puncture could have triggered the reactivation of herpes virus infection (51, 85). In this context. a systematic review on HA filling of nasolabial folds found that the correct injection technique (avoiding fan-like injection) applying slow rate injection (0.3 ml/min) can minimize the risk of injection-related effects (8).

Moreover, our analysis uncovered some AEs that have been reported only in case reports, such as foreign granulomatous reactions with histology (0.6%), tumor-like nodule (0.3%). Angioedema (0.3%) was reported in one RCT (42) and in three case reports (60, 62, 70). Filler-associated necrosis of the lips were noted in three case reports (86–88). The available systematic reviews have not identified such AEs in similar prevalence (3, 8, 17, 80, 89). Additionally, vocalization and mastication may also be disturbed but no reports are available about this.

Impurities in HA could be a potential explanation for immune system-related AEs. HA itself is a non-allergic and a non-toxic molecule (90). However, in health industry products HA is manufactured from various xenogeneic sources. Also, there are differences between the various HA manufacturing procedures (31, 80, 91). In our meta-analysis all included studies used HA produced by bacterial transduction. HA products originating from bacterial transduction, using advanced purification technologies are thought to reduce the risk of host immune response compared to HA products from animal sources (80). Rough HA preparations may be further modified chemically to create the cross-links that extend the lifespan of the injected HA (80, 91). However, impurities, residual proteins and nucleic acid fragments leading to immune reactions may still exist after purification (31, 32, 91). It is unclear whether the few cases of angioedema and granulomas were due to impurities in the used HA products or by the possible contamination of the needle with bacteria used to puncture the skin (92–94). But a recent systematic review investigated the incidence of delayed inflammatory reactions associated with HA injection (17). That work concluded that although the estimated incidence is relatively low, preceding skin tests could be still relevant before HA injection to prevent certain types of granulomas, such as the ones caused by delayed-type hypersensitivity reaction (17).

## Limitations

A major limitation of the present work is the relatively small number of RCTs found on the topic. Although our analysis revealed the importance of confounding factors, no sub-group analysis could be performed due to the limited reported data, and to uncomprehensive data-reporting. For example, the volume of HA injection and the injection technique were not given, and subdivision of the results according to skin types were not always provided. Additionally, different studies used different reporting schemes and wording for detecting AEs. Hence, due to the lack of clear definition, we had to merge certain reported AEs based on our estimation.

In several papers, the documentation of AEs was not optimal for comparison. Additionally, most of the prospective studies had a short follow-up period, up to a maximum of 1 year. Moreover, our analysis was based on reported events, unreported events could not be taken into account. This may cause underestimation of the number and type of AEs associated with lip augmentation.

## Conclusion

In conclusion, our meta-analysis provided evidence that hyaluronic acid injections are highly efficient at least up to 6 months. Even after 1 year following HA injections, in almost half of the patients, the lip volume was still significantly increased. Additionally, we found that most of the AEs after HA treatment were mild or moderate. But the lack of longer follow-ups could not reveal possible delayed reactions. Based on our present meta-analysis, we suggest that more high quality RCTs are needed to strengthen the certainty of evidence and firmly establish the long-term effect of HA injection for lip augmentation.

## Data Availability Statement

The original contributions presented in the study are included in the article/Supplementary Material, further inquiries can be directed to the corresponding author.

## Author Contributions

LC, SF, AL, VG, SK, PH, and GV devised the project, the main conceptual ideas, and planned the research. LC, SF, NG, DC, SK, and GV worked out the methodology. LC, SF, and GV performed the data collection: literature search, study selection, and data extraction. LC, AM, and DC also organized and maintained research data for analysis. NG performed analytic calculations, applied statistical models for synthetizing data, and visualized synthetized data into forest plots. AM, DC, and SK also aided the research by interpretation of raw and synthetized data. LC and SF worked on summarizing results into figures and tables. SK and GV were responsible for managing and coordinating the research activity. PH and GV took leadership responsibility for the research activity, provided resources, and acquired financial support for the research project. SK, PH, and GV validated reproducibility of the results. LC, NG, AM, DC, and GV wrote the manuscript with input from all authors. SF, AL, VG, SK, PH, and GV extensively reviewed the work and further edited the manuscript. All authors contributed to the article and approved the submitted version.

## Conflict of Interest

The authors declare that the research was conducted in the absence of any commercial or financial relationships that could be construed as a potential conflict of interest.

## Publisher's Note

All claims expressed in this article are solely those of the authors and do not necessarily represent those of their affiliated organizations, or those of the publisher, the editors and the reviewers. Any product that may be evaluated in this article, or claim that may be made by its manufacturer, is not guaranteed or endorsed by the publisher.
