# Supplementary material for: Hyaluronic Acid Is an Effective Dermal Filler for Lip Augmentation: A Meta-Analysis
Source: Front Surg. 2021 Aug 6;8:681028. doi: 10.3389/fsurg.2021.681028 (PMC8377277; doi:10.3389/fsurg.2021.681028)
Supplement: Supplementary file 2 [file Table_2.DOCX]

**Supplementary Table 2.** Search query.

| **Database** | **Search query** |
| --- | --- |
| CENTRAL | *“*('hyaluronic':ti, ab, kw OR 'hyaluronate':ti, ab, kw OR 'hyaluronan':ti, ab, kw OR 'dermal filler':ti, ab, kw OR 'injectable implant':ti, ab, kw) AND 'lip':ti, ab, kw.” Limits applied: trials. |
| Embase | “('hyaluronic':ti, ab, kw OR 'hyaluronate':ti, ab, kw OR 'hyaluronan':ti, ab, kw OR 'dermal filler':ti, ab, kw OR 'injectable implant':ti, ab, kw) AND 'lip':ti, ab, kw*”*. Limits applied: human. |
| MEDLINE | *„*hyaluronic[All Fields] OR hyaluronate[All Fields] OR ("hyaluronic acid"[MeSH Terms] OR ("hyaluronic"[All Fields] AND "acid"[All Fields]) OR "hyaluronic acid"[All Fields] OR "hyaluronan"[All Fields]) OR ("dermal fillers"[MeSH Terms] OR ("dermal"[All Fields] AND "fillers"[All Fields]) OR "dermal fillers"[All Fields] OR ("dermal"[All Fields] AND "filler"[All Fields]) OR "dermal filler"[All Fields]) OR (("injections"[MeSH Terms] OR "injections"[All Fields] OR "injectable"[All Fields]) AND implant[All Fields]) AND ("lip"[MeSH Terms] OR "lip"[All Fields]) AND "loattrfull text"[sb]*”.* Limit applied: „human”. |
