# Supplementary material for: Hyaluronic Acid Is an Effective Dermal Filler for Lip Augmentation: A Meta-Analysis
Source: Front Surg. 2021 Aug 6;8:681028. doi: 10.3389/fsurg.2021.681028 (PMC8377277; doi:10.3389/fsurg.2021.681028)
Supplement: Supplementary file 3 [file Table_3.DOCX]

**Supplementary Table 3.** Description of the modified Newcastle Ottawa Scale to suite included study assessment.

| **Selection** | 1) Representativeness of the exposed cohort  a) truly representative of the average subjects for lip augmentation (describe) in the community **🟑**  b) somewhat representative of the average subjects for lip augmentation in the community **🟑**  c) selected group of users eg nurses, volunteers  d) no description of the derivation of the cohort |
| --- | --- |
|  | 2) Selection of the non - exposed cohort  a) drawn from the same community as the exposed cohort **🟑**  b) drawn from a different source  c) no description of the derivation of the non exposed cohort |
|  | 4) Demonstration that outcome of interest was not present at start of study  a) yes **🟑**  b) no |
| **Comparability** | 1) Comparability of cohorts on the basis of the design or analysis  a) study controls for __lip fullness_____ (select the most important factor) **🟑**  b) study controls for any additional factor **🟑** |
| **Outcome** | 1) Assessment of outcome  a) independent blind assessment **🟑**  b) record linkage – *outcome was assessed by non blinded treating investigator using a validated lip fulness scale.***🟑**  c) self report  d) no description  2) Was follow-up long enough for outcomes to occur  a) yes (6 months) **🟑**  b) no  3) Adequacy of follow up of cohorts  a) complete follow up - all subjects accounted for **🟑**  b) subjects lost to follow up unlikely to introduce bias - 90 % follow up, or description provided of those  lost) **🟑**  c) follow up rate < 90% and no description of those lost  d) no statement |
