# Supplementary material for: Hyaluronic Acid Is an Effective Dermal Filler for Lip Augmentation: A Meta-Analysis
Source: Front Surg. 2021 Aug 6;8:681028. doi: 10.3389/fsurg.2021.681028 (PMC8377277; doi:10.3389/fsurg.2021.681028)
Supplement: Supplementary file 4 [file Table_4.DOCX]

**Supplementary Table 4.** Study characteristics of case reports included in adverse reaction analysis.

| **Study** | **Number of participants** | **Female ratio (%)** | **Age or mean age ± SD** | **Treatment** | **Description of symptoms** | **Diagnosis** | **Time after HA treatment** |
| --- | --- | --- | --- | --- | --- | --- | --- |
| Anatelli, F. et al. 2010 (56) | 1 | 100 | 80 | Restylane | 3 mm pearly translucent papule in the left upper cutaneous lip | Biopsy, amorphous basophilic deposit of HA | 8 weeks or more (not clearly stated) |
| Bulam, H. et al. 2015 (57) | 1 | 100 | 27 | Juvederm Ultra Plus XC | Angioedema-type acute hypersensitivity reaction | Clinical signs, angioedema | Within minutes |
| Curi, M. M. et al. 2015 (58) | 2 | 100 | 65 | Restylane | 2 nodular lesions on the right upper lip mucosa | Biopsy, granulomatous foreign body reaction related to the HA | 12 years |
|  |  |  | 58 | HA (unknown) | Sudden symmetric bilateral swelling on the parotid masseteric region and also on the buccal mucosa after chemotherapy | Clinical examination | 4 years |
| Dougherty, A. L. et al. 2011 (59) | 1 | 100 | Unknown | Restylane | Angioedema-type swelling on both lips | Clinical signs, angioedema, herpes reactivation | Within 12 hours |
| Duhovic, C. et al. 2016 (60) | 1 | 100 | 58 | HA (unknown) | Indurated nodules above the upper lip bilaterally | Biopsy, multinucleated giant cells granulomatosis | 4 years |
| Edwards, P. C. et al. 2006 (61) | 1 | 100 | 74 | Restylane | Firm submucosal nodule of the lower lip | Biopsy, multiple cystlike vacuolated areas with histiocytes and foamy macrophages, consistent with a foreign body reaction | 6 months |
| Eversole, R.  et al. 2013 (62) | 2 | 100 | 45 | HA (unknown) | Granular yellow lesion | Biopsy, granulomatous foreign body reaction | Within 5 years |
|  |  |  | 51 | HA (unknown) | White nodules mandibular sulcus |  |  |
| Farahani, S. S. et al. 2012 (63) | 3 | 100 | 56 (mean) | Restylane | Painless discrete nodule on the labial mucosa | Biopsy | Within 2 years |
| Feio, P. S. et al. 2013 (64) | 2 | 100 | 51 | HA (unknown) | Hardness in the lower lip mucosa | Biopsy, presence of numerous giant cells around translucent particles, foreign body reaction | 6 months |
|  |  |  | 30 |  | Fibrous nodule on the left upper lip mucosa, which had quickly enlarged, then stabilised | No surgical intervention | 7 years |
| Fernández-Aceñero Ma, J. et al. 2003 (65) | 1 | 100 | 48 | Restylane | Several discrete nodules in the upper lip | Biopsy, ranulomatous foreign body reaction with multinucleated cells around a blue amorphous material | 2 years |
| Grippaudo, F. R. et al. 2014 (66) | 1 | 100 | 28 | HA (unknown) | “angry red nodules” | High Frequency Ultrasound (HFUS) examination | 1 year |
| Leonhardt, J. M. et al. 2005 (67) | 1 | 100 | 52 | Restylane | Angioedema-type swelling on the upper lips | Clinical signs, angioedema | Within hours |
| Martin, L. et al. 2018 (68) | 2 | 100 | 24 | HA (unknown) | Nodules in the lip area | Biopsy, Foreign body reaction to exogenous material | Within years |
|  |  |  | 43 |  |  |  |  |
| Wolfram, D. et al. 2006 (69) | 1 | 100 | 53 | Restylane | Erythematous  indurations of both nasolabial folds | Dense granulomatous infiltrate with multinucleated giant cells as well as sharply delineated extracellular foreign bodies | 2 years |

Abbreviations: HA: hyaluronic acid.
