# Supplementary material for: Hyaluronic Acid Is an Effective Dermal Filler for Lip Augmentation: A Meta-Analysis
Source: Front Surg. 2021 Aug 6;8:681028. doi: 10.3389/fsurg.2021.681028 (PMC8377277; doi:10.3389/fsurg.2021.681028)
Supplement: Supplementary file 5 [file Table_5.DOCX]

**Supplementary Table 5.** Detailed risk of bias assessment of included RCTs using the Cochrane risk of bias tool.

| **Beer, K. et al. 2015 (38)** | | |
| --- | --- | --- |
| **Bias** | **Author’s judgement** | **Support for judgement** |
| Random sequence generation (selection bias) | Low risk | "Patients were randomly assigned 3:1 to receive small particle [...] HA [...] or no treatment." |
| Allocation concealment (selection bias) | Low risk | "centralized randomization system was used" |
| Blinding of participants and personnel (performance bias) | High risk | Comment: no blinding was applied on the treating investigator and participants. |
| Blinding of outcome assessment (detection bias) | Low risk | "evaluator-blinded study" |
| Incomplete outcome data (attrition bias) | Low risk | "A total of199 patients completed the study (91%). No patient discontinued because of AEs; main reasons were lost to follow-up (6%) and withdrawal of consent (3%)." |
| Selective reporting (reporting bias) | Unclear risk | Comment: No access to study protocol or trial registry entry, but no intext evidence of reporting bias. |
| **Carruthers, A. et al. 2010 (49)** | | |
| **Bias** | **Author’s judgement** | **Support for judgement** |
| Random sequence generation (selection bias) | Low risk | "multicenter (3 site), prospective, single-blind, randomized, parallel-group study..." |
| Allocation concealment (selection bias) | Unclear risk | Comment: no description of method used for allocation concealment. |
| Blinding of participants and personnel (performance bias) | High risk | Comment: no blinding was applied on the treating investigator and participants. |
| Blinding of outcome assessment (detection bias) | Low risk | "an assessing investigator who was masked to the treatment that the subject received conducted effectiveness evaluations." |
| Incomplete outcome data (attrition bias) | High risk | Comment: 23% of participants dropped out of the study group in interest. |
| Selective reporting (reporting bias) | Unclear risk | Comment: No access to study protocol or trial registry entry, but no intext evidence of reporting bias. |
| **Dayan, S. et al. 2015 (39)** | | |
| **Bias** | **Author’s judgement** | **Support for judgement** |
| Random sequence generation (selection bias) | Low risk | "Block randomization (i.e., randomization within subgroups) was performed" |
| Allocation concealment (selection bias) | Unclear risk | Comment: Not mentioned in publication. |
| Blinding of participants and personnel (performance bias) | High risk | Comment: no blinding was applied on the treating investigator and participants. |
| Blinding of outcome assessment (detection bias) | Low risk | "the blinded evaluating investigator" |
| Incomplete outcome data (attrition bias) | Unclear risk | "One subject in the treatment group discontinued because of an AE; all other discontinuations were due to withdrawn consent, lost to follow-up, or other. One subject in the control group who subsequently received treatment discontinued because of pregnancy; all other discontinuations were due to withdrawn consent, lost to follow-up, or other." Comment: "other" is not specified. |
| Selective reporting (reporting bias) | Low risk | Comment: Protocol is available, and all pre-specified outcomes are reported. |
| **Downie, J. et al. 2009 (72)** | | |
| **Bias** | **Author’s judgement** | **Support for judgement** |
| Random sequence generation (selection bias) | Low risk | "Patients [...] were randomized by using a computerized Interactive Voice Response (IVR) system." |
| Allocation concealment (selection bias) | Low risk | "...patients assigned to PRI 1, PRI 2 or Perlane treatments received a similar saline skin test." & "The IVR was accessed using a push button telephone; the system provided both randomization and unblinding facility for the study." |
| Blinding of participants and personnel (performance bias) | Unclear risk | Comment: Participants were blinded, but personnel injecting dermal fillers were not. |
| Blinding of outcome assessment (detection bias) | Low risk | "Patients were assessed post-operatively [...] by an independently qualified blinded assessor." |
| Incomplete outcome data (attrition bias) | Low risk | "Seventy-nine patients were enrolled into the study: PRI 1: 19 patients, PRI 2: 19 patients, Perlane: 23 patients, Zyplast: 18 patients, one patient dropped out of the study." |
| Selective reporting (reporting bias) | Unclear risk | Comment: No evidence found. |
| **Geronemus, R. G. et al. 2017 (40)** | | |
| **Bias** | **Author’s judgement** | **Support for judgement** |
| Random sequence generation (selection bias) | Low risk | "subjects were randomized 3:1 to treatment with VYC-15LorNASHA" |
| Allocation concealment (selection bias) | Low risk | "Subjects were blinded to treatment assignment, which was based on a central block randomization schedule and an automated interactive voice/web response system." |
| Blinding of participants and personnel (performance bias) | Unclear risk | "Subjects were blinded to treatment" & "unblinded treating investigator" |
| Blinding of outcome assessment (detection bias) | Low risk | "blinded evaluating investigator" |
| Incomplete outcome data (attrition bias) | Low risk | "One subject assigned to VYC-15L discontinued before receiving treatment." |
| Selective reporting (reporting bias) | Low risk | Comment: Protocol is available, and all pre-specified outcomes are reported. |
| **Glogau, R. G. et al. 2012 (41)** | | |
| **Bias** | **Author’s judgement** | **Support for judgement** |
| Random sequence generation (selection bias) | Low risk | "randomly assigned 3:1 to receive SGPHA or no treatment" |
| Allocation concealment (selection bias) | Low risk | "centralized randomization system was used" |
| Blinding of participants and personnel (performance bias) | High risk | Comment: no blinding was applied on the treating investigator and participants. |
| Blinding of outcome assessment (detection bias) | Low risk | "evaluator-blinded study" |
| Incomplete outcome data (attrition bias) | Low risk | "One hundred sixteen (86%) in the SGP-HA group and 39 (87%) in the no-treatment group completed the study." |
| Selective reporting (reporting bias) | Unclear risk | Comment: No access to study protocol or trial registry entry, but no intext evidence of reporting bias. |
| **Raspaldo, H. et al. 2015 (42)** | | |
| **Bias** | **Author’s judgement** | **Support for judgement** |
| Random sequence generation (selection bias) | Low risk | "subjects were randomized (1:1) to receive Juvéderm Volbella [...] or Restylane-L" |
| Allocation concealment (selection bias) | Low risk | "randomization and treatment assignment were managed by a centralized, automated, interactive voice and Web response system" |
| Blinding of participants and personnel (performance bias) | Unclear risk | "Subjects, independent central reviewers and investigational staff except for investigators and study coordinators remained blinded to treatment assignment." |
| Blinding of outcome assessment (detection bias) | Low risk | "Subjects, independent central reviewers and investigational staff except for investigators and study coordinators remained blinded to treatment assignment." |
| Incomplete outcome data (attrition bias) | Low risk | "Most subjects completed the study: 118 (84.9%) in the Juvéderm Volbella with Lidocaine group and 115 (81.0%) in the Restylane - L group." |
| Selective reporting (reporting bias) | Low risk | Comment: Protocol is available, and all pre-specified outcomes are reported. |
