# Supplementary material for: Hyaluronic Acid Is an Effective Dermal Filler for Lip Augmentation: A Meta-Analysis
Source: Front Surg. 2021 Aug 6;8:681028. doi: 10.3389/fsurg.2021.681028 (PMC8377277; doi:10.3389/fsurg.2021.681028)
Supplement: Supplementary file 6 [file Table_6.DOCX]

**Supplementary Table 6.** Detailed assessment of risk of bias of cohort studies using the Newcastle-Ottawa Scale.

| Study | Selection | | | | Comparability | Outcome | | | Score (max 7) |
| --- | --- | --- | --- | --- | --- | --- | --- | --- | --- |
|  | Representative-ness of the intervention group | Selection of the non-exposed cohort | Ascertainment of intervention | Demonstration that outcome of interest was not present at start of the study | Comparability | Assessment of outcome | Was follow-up long enough for outcome to occur? | Adequacy of follow up of cohorts |  |
| Chopra, R. et al. 2018 (43) | a) | a) | N/A | a) | Baseline-controlled | b) | 3 months | 3 dropouts (95% completed) | 6 |
| Eccleston, D. et al. 2012 (44) | a) | a) | N/A | a) | Baseline-controlled | b) | 12 months, primary effect endpoint at 3 months | 1 dropout (98.3% completed) | 6 |
| Fagien, S. et al. 2013 (45) | a) | a) | N/A | a) | Baseline-controlled | b) | 6 months | No dropout | 7 |
| Solish, N. et al 2011 (46) | b) | a) | N/A | a) | Baseline-controlled | a) | 2 months | 3 dropouts (85.7% completed) | 6 |
| Yazdanparast, T. et al. 2017 (47) | b) | a) | N/A | a) | Baseline-controlled | a) | 6 months | No dropout | 7 |
|  |  |  |  |  |  |  |  |  |  |
| Artzi, O. et al. 2016 (50) | a) | a) | N/A | a) | No control group | d) | 11 months | d) | 5 |
| Carruthers, J. et al 2005 (51) | b) | a) | N/A | a) | No control group | c) | 6 months | 1 drop out (94% completed) | 6 |
| Fischer, T. et al. 2016 (52) | b) | a) | N/A | a) | No control group | b) | 4 months | d) | 5 |
| Philipp-Dormston, W. G. et al. 2014 (53) | b) | a) | N/A | a) | No control group | c) | 1 months | d) | 4 |
| Rzany, B. et al. 2012 (54) | b) | a) | N/A | a) | No control group | b) | 6 months | 1 drop out (98% completed) | 7 |
| Samuelson, U. et al. 2015 (55) | b) | a) | N/A | a) | Baseline-controlled | c) | 9 months | 1 drop out (96% completed) | 6 |

Abbreviations: N/A: not applicable.
