# Supplementary material for: Hyaluronic Acid Is an Effective Dermal Filler for Lip Augmentation: A Meta-Analysis
Source: Front Surg. 2021 Aug 6;8:681028. doi: 10.3389/fsurg.2021.681028 (PMC8377277; doi:10.3389/fsurg.2021.681028)
Supplement: Supplementary file 7 [file Table_7.DOCX]

**Supplementary Table 7.** Summary of the risk of bias assessment of cohort studies.

| **Study** | **Selection** | **Comparability** | **Outcome** | **Total Score** | **Included in effectiveness analysis** |
| --- | --- | --- | --- | --- | --- |
| Chopra, R. et al. 2018 (43) | **🟑🟑🟑** | **🟑** | **🟑🟑** | 6 | Yes |
| Eccleston, D. et al. 2012 (44) | **🟑🟑🟑** | **🟑** | **🟑🟑** | 6 | Yes |
| Fagien, S. et al. 2013 (45) | **🟑🟑🟑** | **🟑** | **🟑🟑🟑** | 7 | Yes |
| Solish, N. et al 2011 (46) | **🟑🟑🟑** | **🟑** | **🟑🟑** | 6 | Yes |
| Yazdanparast, T. et al. 2017 (47) | **🟑🟑🟑** | **🟑** | **🟑🟑🟑** | 7 | Yes |
| Artzi, O. et al. 2016 (50) | **🟑🟑🟑** | **-** | **🟑** | 4 | No |
| Carruthers, J. et al 2005 (51) | **🟑🟑🟑** | **-** | **🟑🟑** | 5 | No |
| Fischer, T. et al. 2016 (52) | **🟑🟑🟑** | **-** | **🟑** | 4 | No |
| Philipp-Dormston, W. G. et al. 2014 (53) | **🟑🟑🟑** | **-** | - | 3 | No |
| Rzany, B. et al. 2012 (54) | **🟑🟑🟑** | **-** | **🟑🟑🟑** | 6 | No |
| Samuelson, U. et al. 2015 (55) | **🟑🟑🟑** | **🟑** | **🟑🟑** | 6 | No |
