# Supplementary material for: Hyaluronic Acid Is an Effective Dermal Filler for Lip Augmentation: A Meta-Analysis
Source: Front Surg. 2021 Aug 6;8:681028. doi: 10.3389/fsurg.2021.681028 (PMC8377277; doi:10.3389/fsurg.2021.681028)
Supplement: Supplementary file 8 [file Table_8.DOCX]

**Supplementary Table 8.** Summary of adverse effects reported in included studies.

| **Adverse effect** | **N**  **(total = 1487)** | **%** |  | **Adverse effect** | **N**  **(total = 1487)** | **%** |
| --- | --- | --- | --- | --- | --- | --- |
| Tenderness | 1320 | 88.7 |  | A tumorlike nodule | 4 | 0.3 |
| Injection site swelling | 1105 | 74.3 |  | Angioedema | 4 | 0.3 |
| Contusion | 725 | 48.7 |  | Dry lip | 3 | 0.2 |
| Injection site mass | 406 | 27.3 |  | Anesthesia | 1 | 0.1 |
| Injection site pain | 293 | 19.7 |  | Canker sore | 1 | 0.1 |
| Erythema | 108 | 7.3 |  | Induration | 1 | 0.1 |
| Tyndall effect and discoloration | 84 | 5.7 |  | Inflammatory nodules | 1 | 0.1 |
| Hematoma | 27 | 1.8 |  | Injection site cyst | 1 | 0.1 |
| Lip disorder | 12 | 0.8 |  | Hemorrhage | 1 | 0.1 |
| Granulomatous foreign body reaction | 9 | 0.6 |  | Papule | 1 | 0.1 |
| Paresthesia | 9 | 0.6 |  | Presyncope | 1 | 0.1 |
| Herpes labialis | 9 | 0.6 |  |  |  |  |
