# Supplementary material for: Hyaluronic Acid Is an Effective Dermal Filler for Lip Augmentation: A Meta-Analysis
Source: Front Surg. 2021 Aug 6;8:681028. doi: 10.3389/fsurg.2021.681028 (PMC8377277; doi:10.3389/fsurg.2021.681028)
Supplement: Supplementary file 9 [file Table_9.DOCX]

**Supplementary Table 9.** Certainty of evidence point according to GRADE approach.

| **Outcome** | **Study design**  **[№ of studies]** | **Initial level of evidence** | **Evidence components** | **Upgrade/ downgrade of evidence** | **Comment** | **Final level of evidence** |
| --- | --- | --- | --- | --- | --- | --- |
| Rate of responders | RCT  [5] (38-42)  Cohort studies  [5] (43-47) | Low | **Risk of bias** | Considerable | Control groups are not used in each study | 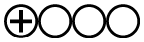  Very Low |
|  |  |  | **Inconsistency** | Serious | Large I^2^ value |  |
|  |  |  | **Indirectness** | Not serious | - |  |
|  |  |  | **Imprecision** | Serious | Wide range of CIs |  |
|  |  |  | **Other considerations** | Publication bias suspected | Small study effect |  |
| Adverse effects | RCT  [6] (38-40, 42, 49, 72)  Cohort studies  [11] (43-47, 50-55)  Case reports  [14] (56-69) | Low | **Risk of bias** | Considerable | Control groups are not used in each study | 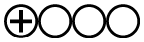  Very Low |
|  |  |  | **Inconsistency** | Serious | Lack of consistent reporting of adverse effects |  |
|  |  |  | **Indirectness** | Not serious | - |  |
|  |  |  | **Imprecision** | Not serious | - |  |
|  |  |  | **Other considerations** | Upgrade by one point | Large effect |  |

Abbreviations: RCT: randomized controlled trial
