# Supplementary figures and images for: Hyaluronic Acid Is an Effective Dermal Filler for Lip Augmentation: A Meta-Analysis
Source: Front Surg. 2021 Aug 6;8:681028. doi: 10.3389/fsurg.2021.681028 (PMC8377277; doi:10.3389/fsurg.2021.681028)

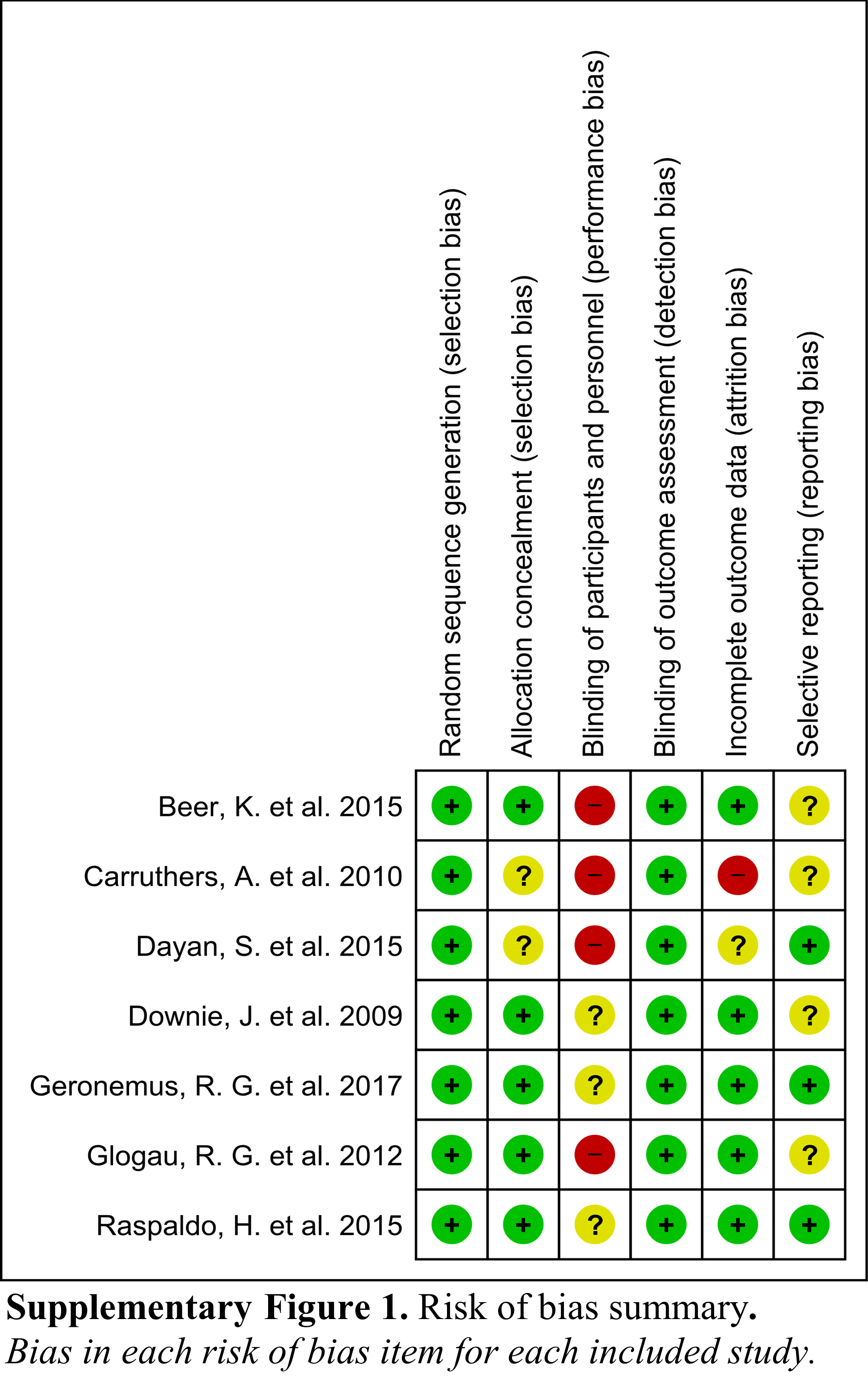

Supplement: Supplementary file 10 [file Image_1.TIF]

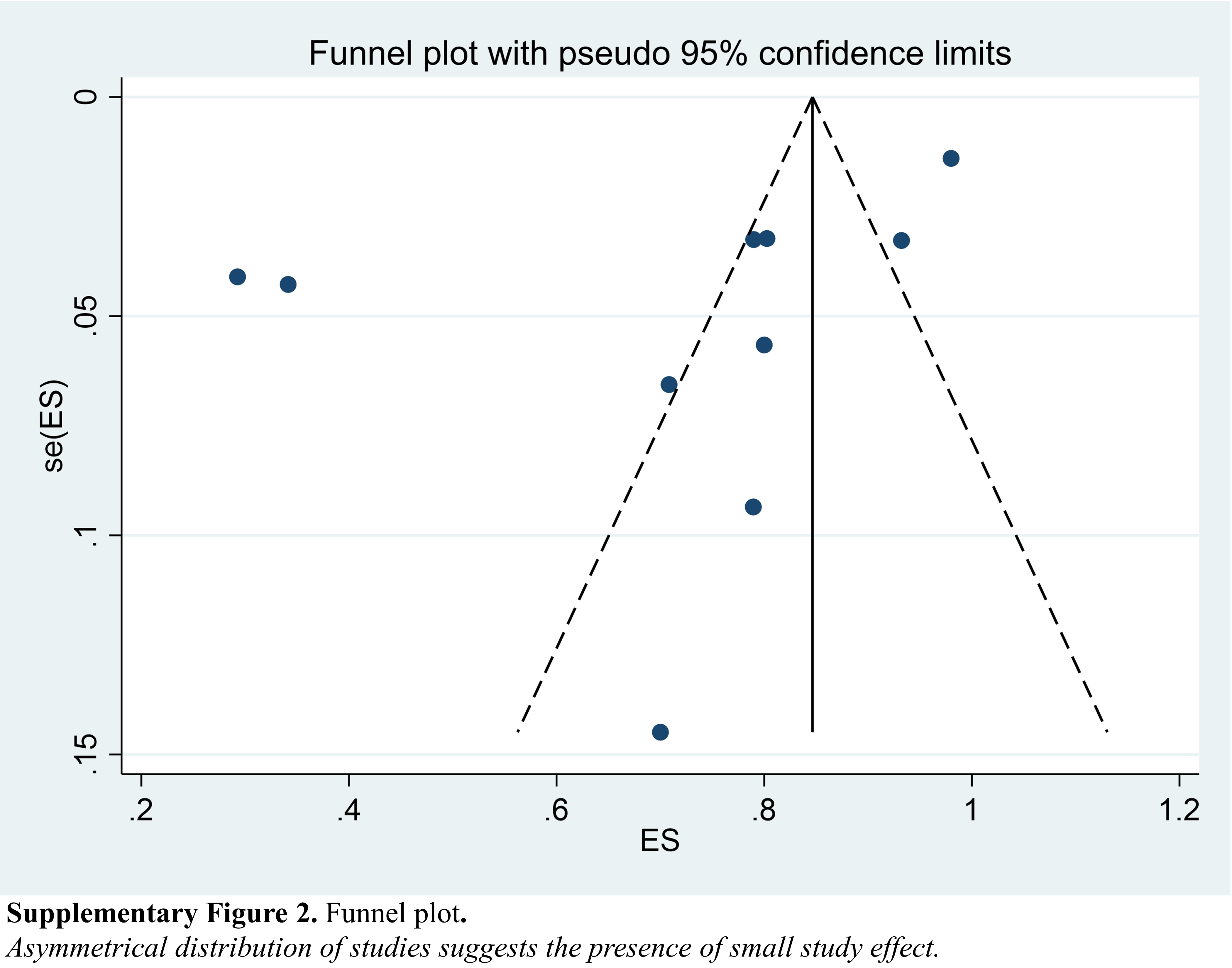

Supplement: Supplementary file 11 [file Image_2.TIF]
